# A novel Fc-engineered human ICAM-1/CD54 antibody with potent anti-myeloma activity developed by cellular panning of phage display libraries

**DOI:** 10.18632/oncotarget.20641

**Published:** 2017-09-05

**Authors:** Katja Klausz, Michael Cieker, Christian Kellner, Hans-Heinrich Oberg, Dieter Kabelitz, Thomas Valerius, Renate Burger, Martin Gramatzki, Matthias Peipp

**Affiliations:** ^1^ Division of Stem Cell Transplantation and Immunotherapy, Department of Medicine II, University Hospital Schleswig-Holstein and Christian-Albrechts-University Kiel, Kiel, Germany; ^2^ Institute of Immunology, University Hospital Schleswig-Holstein and Christian-Albrechts-University Kiel, Kiel, Germany

**Keywords:** multiple myeloma, immunotherapy, antibody, ICAM-1, phage display

## Abstract

To identify antibodies suitable for multiple myeloma (MM) immunotherapy, a cellular screening approach was developed using plasma cell lines JK-6L and INA-6 and human synthetic single-chain fragment variable (scFv) phage libraries. Isolated phage antibodies were screened for myeloma cell surface reactivity. Due to its binding characteristics, phage PIII-15 was selected to generate the scFv-Fc fusion protein TP15-Fc with an Fc domain optimized for FcγRIIIa binding. Various MM cell lines and patient-derived CD138-positive malignant plasma cells, but not granulocytes, B or T lymphocytes from healthy donors were recognized by TP15-Fc. Human intercellular adhesion molecule-1 (ICAM-1/CD54) was identified as target antigen by using transfected Chinese hamster ovary (CHO) cells. Of note, no cross-reactivity of TP15-Fc with mouse ICAM-1 transfected cells was detected. TP15-Fc was capable to induce antibody-dependent cell-mediated cytotoxicity (ADCC) against different human plasma cell lines and patients’ myeloma cells with peripheral blood mononuclear cells (PBMC) and purified NK cells. Importantly, TP15-Fc showed potent *in vivo* efficacy and completely prevented growth of human INA-6.Tu1 plasma cells in a xenograft SCID/beige mouse model. Thus, the novel ADCC-optimized TP15-Fc exerts potent anti-myeloma activity and has promising characteristics to be further evaluated for MM immunotherapy.

## INTRODUCTION

MM is a malignant plasma cell disorder that accounts for approx. 10-15% of the hematologic malignancies in the US and Europe [1, 2]. So called ‘novel drugs’ like proteasome inhibitors (PIs) and immunomodulatory drugs (IMiDs) in combination with stem cell transplantation have led to an increased overall survival [2, 3], but still most of the patients, especially patients ineligible for transplantation, older than 65 years and/or relapsed/refractory to PIs and IMiDs, succumb to their disease and new treatment approaches are needed. In recent years, more and more efforts have been made to evaluate monoclonal antibodies (mAbs) and antibody-derived immunotherapeutic agents for use in MM therapy [4–7]. As first-in-class agents daratumumab (CD38) and elotuzumab (CD319) have been FDA-approved at the end of 2015 [8, 9].

Therapeutic mAbs are well established for the treatment of hematologic malignancies and solid tumors [10, 11]. To date, they are predominantly of human IgG1 isotype. IgG1 mAbs exert their functions either directly via Fab-mediated effects or by interacting with certain Fcγ receptors (FcγR) on immune cells as well as by activating complement. Since the FcγRIIIa V158F and the FcγRIIa H131R polymorphisms were correlated with the ability of IgG1 mAbs to efficiently recruit immune effector cells, i.e. NK cells for ADCC and macrophages for ADCP, and have an impact on their clinical efficacy, strategies have been developed to improve effector cell recruitment [12, 13]. Such attempts include amino acid exchanges (protein-engineering) and modifications in the glycosylation pattern (glyco-engineering) [14–16]. Currently, two glyco-engineered mAbs, obinutuzumab (CD20) and mogamulizumab (anti-CCR4), are approved as anti-cancer agents [17, 18], and protein-engineered mAbs are evaluated in preclinical studies [19–21]. For MM therapy, such engineered mAbs might be beneficial for instance in the post-transplant setting where NK cells recover early after stem cell transplantation [22, 23], or in full-blown MM where NK cell numbers and activity are also low [24]. Moreover, the combination of Fc-engineered mAbs with lenalidomide, known to lower NK cell activation levels and to stimulate immune effector cells [25, 26], can be a promising approach. As shown for Xmab5592, a humanized IgG1 directed against HM1.24/CD317, Fc-engineered mAbs can have potent anti-myeloma activity and be synergistically active in combination with lenalidomide *in vivo* [27].

In addition, anti-myeloma agents that impair interactions between the bone marrow (BM) microenvironment and malignant plasma cells can be of particular interest [28]. Cell surface proteins which are involved in myeloma cell adhesion to BM stromal cells (BMSC) could be potential targets for therapeutic mAbs. Those include members of the integrin and adhesion protein families and their natural receptors, e.g. vascular cell adhesion molecule-1 (VCAM-1) and intercellular adhesion molecule-1 (ICAM-1/CD54). Increased serum levels of both, VCAM-1 and ICAM-1, were reported to be associated with advanced disease and poor outcome in MM patients [29].

To identify antibodies targeting cell surface antigens on malignant plasma cells that have potential as immunotherapeutic agents, we have employed phage display technology with human single chain fragment variable (scFv) antibody libraries and a cellular panning strategy. Phage PIII-15 was selected based on its favorable binding profile and converted into a human scFv-Fc fusion protein named TP15-Fc, that specifically targets human ICAM-1/CD54. TP15-Fc induced significant ADCC against myeloma cells and, importantly, completely prevented MM growth *in vivo*. Thus, TP15-Fc has potential as a novel therapeutic anti-myeloma antibody.

## RESULTS

### Isolation of phages preferentially binding to myeloma cells

To identify anti-myeloma antibodies, the two human Tomlinson phage libraries were subjected to cellular screening (Supplementary Figure 1). The Tomlinson I and J libraries were generated at Greg Winter's lab in Cambridge and are both based on the most common human frameworks for VH (V3-23/DP-47and JH4b) and Vκ (O12/O2/DPK9 and Jκ1). The high diversity of more than 100 million different scFv fragments/phages per library was reached by side chain diversity incorporated in the antigen binding sites, i.e. the CDR2 and CDR3 regions, through DVT codon usage in the Tomlinson I library and NNK codon usage in the Tomlinson J library. Both libraries were pre-absorbed in consecutive rounds against polymorphonuclear cells (PMN)/granulocytes and T lymphocytes from different healthy donors to deplete phages binding to these leukocyte subsets and HLA antigens. The pre-absorbed libraries were incubated with a mixture of JK-6L MM cells and T lymphocytes. T cells were subsequently depleted by MACS and the vital myeloma cells (CD138^+^/7-AAD^-^) were sorted by FACS. Bound phages were eluted, amplified and used for two additional panning rounds with INA-6 and JK-6L plasma cells. While less than 1×10^4^ phages were isolated after the first panning round from both libraries, panning rounds 2 and 3 resulted in 1-2×10^8^ phages. Enrichment factors (EF) of 7857 and 1333, respectively, were achieved for the Tomlinson I and J libraries from round 1 to 2, while no significant further enrichment was observed with subsequent rounds (Supplementary Table 1).

**Figure 1:**
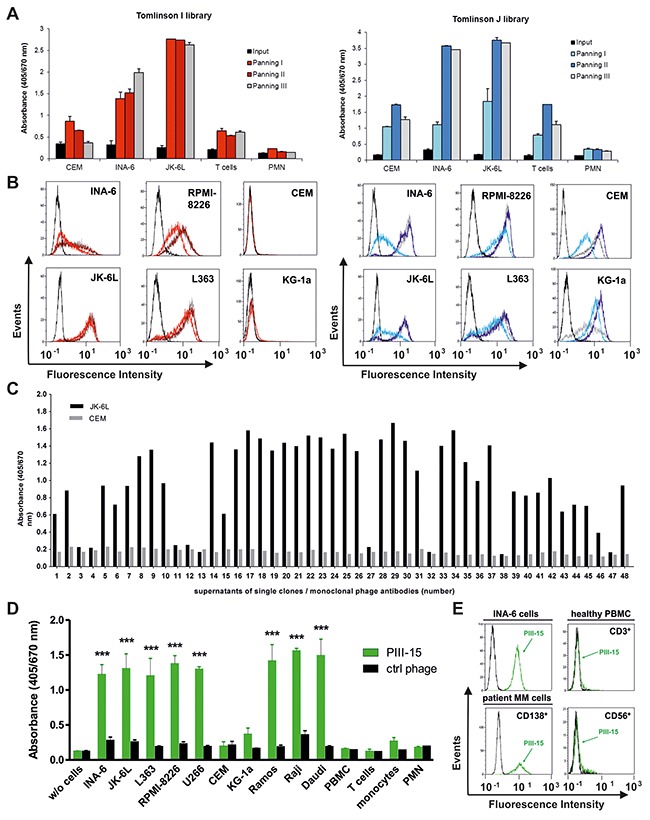
Binding characteristics of phages after panning All cellular ELISA and flow cytometry experiments were performed with 0.5×10^6^ cells per sample. Bound phages were either detected with a FITC-labeled anti-fd bacteriophage antibody (flow cytometry) or with an HRP-labeled anti-M13 antibody (ELISA). **(A)** 2.5×10^11^ phages from the original (input) or the panned libraries from round 1 to 3 (Panning I to III) were incubated with the indicated cells and binding was tested in whole-cell ELISA. Mean values ± SEM from duplicates are given. **(B)** Flow cytometric analyses of phages from Tomlinson I (left panel) and J library (right panel) prior to panning (black lines) and from panning rounds 1 (light red and blue line, respectively), 2 (dark red and blue line, respectively), and 3 (grey lines) with myeloma (INA-6 and JK-6L) and leukemia cell lines (CEM and KG-1a) are shown. **(C)** Binding of monoclonal phage antibody-containing *E. coli* TG1 supernatants (100 μl each) from Tomlinson I library, panning round 3, was tested in ELISA experiments with JK-6L and CEM cells. **(D)** Binding characteristics of the monoclonal phage antibody PIII-15 (5×10^10^ phages) were evaluated by whole-cell ELISA. Mean values ± SEM from three independent experiments with duplicates are given. (***) p < 0.001 mean absorbance of PIII-15 *vs.* ctrl phage. **(E)** Flow cytometric experiments were performed using INA-6, primary patient cells (7-AAD^-^/CD138^+^; frozen sample from a plasma cell leukemia patient with 86 % malignant plasma cells) and PBMC subsets of a healthy donor (7-AAD^-^ and CD3^+^ or CD56^+^). Binding of 5×10^10^ PIII-15 phages (green line) and non-binding control phages (black line) is shown.

The binding properties of the isolated phages from the individual panning rounds were tested by whole-cell ELISA and flow cytometry. As shown for the original libraries (input), no significant binding to any of the tested cell types was observed. In contrast, the phages from the subsequent panning rounds showed increased binding to INA-6, JK-6L, L363, and RPMI-8226 plasma cell lines (Figures 1A and 1B). While granulocytes/PMN were only marginally recognized by all phages, reactivity with T lymphocytes and CEM cells (T-ALL) was detected predominately for the phages of the Tomlinson J library (Figure 1A). Importantly, no reactivity of the Tomlinson I library phages was seen with CEM and the myeloid cell line KG-1a by flow cytometry (Figure 1B). Thus, further analyses were particularly performed with phages from the Tomlinson I library. As shown in Figure 1C, 39 of 48 (81 %) *E. coli* supernatants containing single phage antibodies tested with JK-6L and CEM cells in ELISA showed strong and exclusive reactivity with the JK-6L MM cells. Hence, the applied panning strategy resulted in the successful isolation of monoclonal phage antibodies binding to myeloma cell lines. Of note, since a fixed volume (100 μl) of phage-containing supernatants without prior quantification were used in this ELISA experiment, no direct comparison between the binding properties of the single phage antibodies can be made. By screening defined quantities of 5×10^10^ single phage clones from panning rounds 2 and 3, phage PIII-15, obtained from the third round of panning, was selected for further functional analyses due to its significant binding to different myeloma/plasma cell leukemia (PCL) and Burkitt's lymphoma cell lines, while binding to other leukemia cell lines (CEM, KG-1a), PBMC and the indicated leukocyte subpopulations was not observed (Figure 1D). Importantly, PIII-15 also bound to CD138^+^ malignant plasma cells of a PCL patient, whereas no reactivity was observed with CD3^+^ T lymphocytes and CD56^+^ cells (predominantly NK cells) of an healthy individual (Figure 1E).

### Generation of the human scFv-Fc fusion protein TP15-Fc

**Figure 2:**
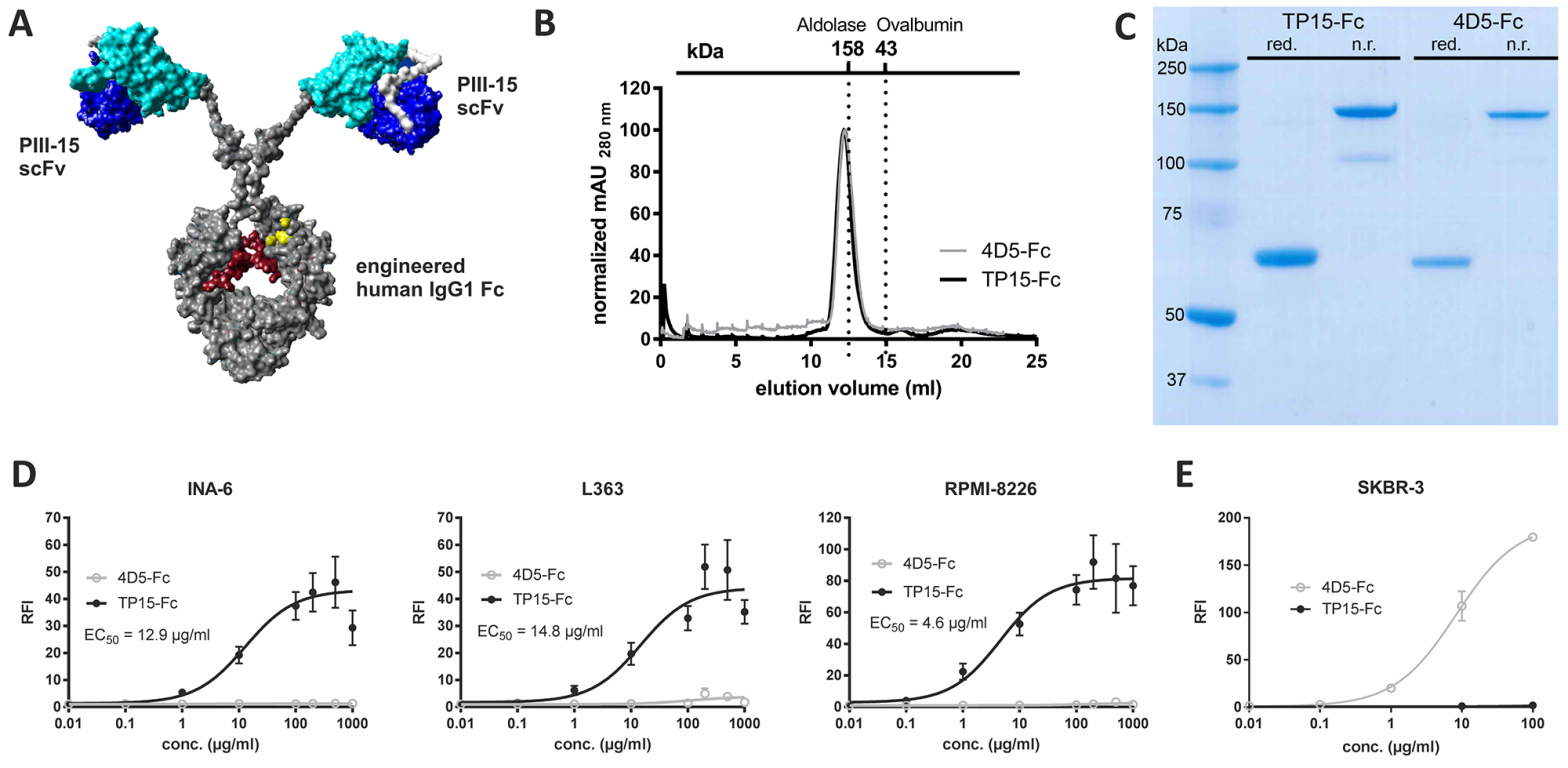
Production of the TP15-Fc **(A)** Homology model of the recombinant scFv-Fc fusion protein consisting of two scFv which were genetically fused to an ADCC-optimized human IgG1 Fc domain (grey) with the introduced mutations (S239D/A330L/I332E) depicted in yellow and the sugar residues in red. Variable heavy and light chains of the PIII-15 scFv are shown in dark and light blue, respectively, with the linker in white. **(B)** Size exclusion chromatography after protein purification by protein A and Ni-NTA affinity chromatography of 200 μg TP15-Fc (black line) and 4D5-Fc (grey line), respectively. **(C)** Coomassie gel with reduced (red.) and non-reduced (n.r.) samples (5 μg protein/lane) from the final preparations. **(D)** Flow cytometric analyses of TP15-Fc (●) revealed dose-dependent binding to INA-6, L363 and RPMI-8226 plasma cell lines. 4D5-Fc (○) was used as a control in these experiments, while its binding to Her2-positive SKBR-3 cells proved functionality of the control molecule **(E)**. Bound antibodies were detected with a FITC-labeled anti-human Fcγ-specific antibody. Graphs show relative fluorescence intensity (RFI) values ± SEM of at least four independent experiments (except n=2 for 4D5-Fc).

The scFv sequence of phage PIII-15 was used to generate a fully human scFv-Fc fusion protein with an ADCC-optimized human IgG1 Fc domain (Figure 2A). By incorporating the amino acid exchanges S239D and I332E in the Fc domain ADCC activity is strongly enhanced [11], whereas the A330L mutation considerably reduces CDC activity of the resulting Fc-engineered scFv-Fc fusion protein TP15-Fc. The recombinant proteins TP15-Fc and the equally constructed control molecule 4D5-Fc, directed against HER2 [57], were expressed by transient transfection of Lenti-X 293T cells and purified from cell culture supernatants by affinity chromatography. Analyses of the protein preparations by size exclusion chromatography revealed a predominant peak at ~12 ml elution volume for both proteins (Figure 2B). Compared to standard proteins, i.e. aldolase (158 kDa) and ovalbumin (43 kDa), TP15-Fc and 4D5-Fc elute in the range of proteins with a molecular weight of ~160 kDa. Gel electrophoresis and Coomassie staining of the final preparations showed pure proteins with molecular masses of ~60 and ~150 kDa under reducing and non-reducing conditions, respectively (Figure 2C). The calculated masses of TP15-Fc and 4D5-Fc were 124 kDa and 128 kDa, respectively. The differences between calculated and observed molecular masses might be due to protein glycosylation and the c-*myc*-(His)_6_-tag which may change migration behaviour in affinity chromatography and gel electrophoresis. Routinely 2.5 - 3.0 mg protein per liter cell culture supernatant was produced. Importantly, concentration-dependent binding of TP15-Fc to INA-6, L363, and RPMI-8226 cells could be measured by flow cytometry, while the control molecule 4D5-Fc did not show any reactivity with these cells (Figure 2D), but as expected, significantly bound to Her2-positive SKBR-3 cells (Figure 2E). The EC_50_ values measured for TP15-Fc binding to the plasmacytoma cell lines were in the range of 37 - 119 nM (4.6 - 14.8 μg/ml).

### TP15-Fc binds to ICAM-1 on myeloma cell lines and malignant plasma cells from patients

**Figure 3:**
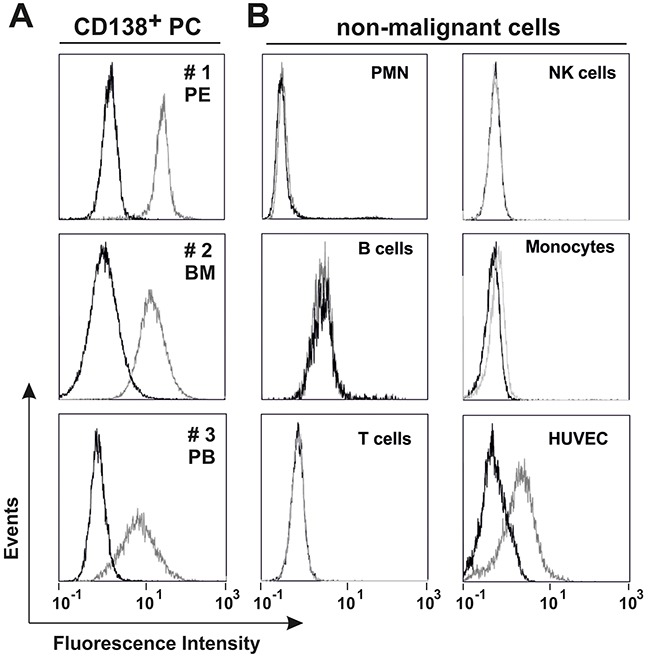
Binding properties of TP15-Fc Flow cytometric analyses of 20 μg/ml 4D5-Fc (black line) or TP15-Fc (grey line) with **(A)** patient-derived malignant plasma cells (PC) from pleural effusion (PE; approx. 78 % CD138^+^ cells), bone marrow (BM; approx. 66 % CD138^+^ cells) or peripheral blood (PB; approx. 91 % CD138^+^ cells) and **(B)** non-malignant cells from healthy donors. FcR on immune cells were blocked with an excess of human Ig (Intratect) prior to incubation with the fusion proteins. An anti-His Alexa Fluor 488 Conjugate was used for detection.

To further investigate the binding specificity of TP15-Fc, flow cytometric analyses were performed with a panel of human lymphoid and non-lymphoid cell lines. As summarized in Table 1, TP15-Fc did neither bind to T-ALL cell lines (CEM, Jurkat) nor to non-lymphoid leukemia cell lines (HEL, KG-1a, HL60, K562), but reacted with different myeloma and Burkitt's lymphoma cell lines. Importantly, TP15-Fc also bound to freshly isolated or cryo-conserved malignant plasma cells from patients with MM and PCL (Figure 3A). Thus, the antigen recognized by TP15-Fc is also expressed on the surface of patients’ MM cells. Reactivity of TP15-Fc with non-malignant leukocytes was tested with isolated subpopulations from healthy donors. TP15-Fc did not bind to granulocytes (PMN), B or T lymphocytes, and showed marginal reactivity with resting NK cells and monocytes. Endothelial cells (HUVEC) were positively stained by TP15-Fc, but antigen density was remarkably lower than that on myeloma cell lines (Figure 3B and Supplementary Figure 4A).

**Table 1:** TP15-Fc binding to human lymphoma and leukemia cell lines

Cell line		Binding	RFI ± SD
**B cell derived**			
INA-6	plasma cell leukemia	++	15.2 ± 5.9
JK-6L	multiple myeloma	+	5.9 ± 0.2
L363	plasma cell leukemia	++	9.8 ± 2.6
MM1.S	multiple myeloma	+	2.7 ± 1.7
RPMI-8226	multiple myeloma	+++	46.2 ± 0.8
U266	multiple myeloma	++	25.8 ± 9.8
ARH-77	B-lymphoblastoid cells (EBV^+^)	+	7.9 ± 2.2
SEM	B cell precursor leukemia	-	1.0 ± 0.1
Daudi	Burkitt's lymphoma	++	10.2 ± 2.1
Raji	Burkitt's lymphoma	++	17.2 ± 7.2
Ramos	Burkitt's lymphoma	+	6.0 ± 1.9
**T cell derived**			
CEM	T-ALL	-	1.0 ± 0.2
Jurkat	T-ALL	-	1.0 ± 0.1
**Non-lymphoid**			
HEL	erythroleukemia	-	1.2 ± 0.2
KG-1a	immature AML	-	1.5 ± 0.6
HL60	AML	-	1.0 ± 0.2
K562	CML	(+)	2.2 ± 0.5

RFI values calculated for binding of 10 μg/ml TP15-Fc relative to 4D5-Fc on 500.000 cells/sample. SD, standard deviation; EBV, Epstein-Barr virus; T-ALL, T-cell acute lymphoblastic leukemia; AML, acute myeloid leukemia; CML, chronic myeloid leukemia; +++ RFI > 30, ++ RFI > 10, + RFI > 2.5, - RFI < 2.

**Figure 4:**
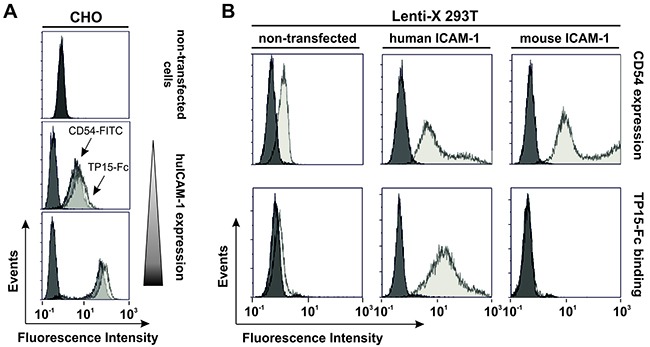
TP15-Fc specifically binds human, but not mouse ICAM-1 **(A)** Flow cytometric analyses of non-transfected and stably transfected CHO-K1 cells with varying ICAM-1 expression levels detected by CD54-FITC (Beckman Coulter; dark grey) correlated with binding of 10 μg/ml TP15-Fc (light grey). 4D5-Fc was used as control (black). **(B)** ICAM-1 expression of non-transfected (upper row, left) and human ICAM-1 transfected Lenti-X 293T cells (upper row, middle) was measured by flow cytometry using mouse anti-human CD54-FITC (grey) and a FITC-labeled isotype control (black). Mouse ICAM-1 was expressed as GFP-tagged protein and positive cells were detected by fluorescence (upper row, right; grey histogram). Non-transfected cells served as control (black). Binding of 100 μg/ml TP15-Fc (grey) and 4D5-Fc (black), respectively, on non-transfected and ICAM-1-positive cell populations was detected with a PE-labeled anti-human Fcγ-specific secondary antibody (lower panel).

Since immunoprecipitation with TP15-Fc failed and the scFv-Fc fusion protein was also not suitable as a detection antibody in Western blotting (data not shown), ELISA and flow cytometric analyses were performed with stably transfected CHO-K1 cells expressing one of nine individual antigens known to be expressed on malignant plasma cells (Supplementary Figure 2). Based on its reactivity with CHO cells expressing CD54, human ICAM-1 was identified as target antigen of TP15-Fc. As shown in Figure 4A, fluorescence intensity of TP15-Fc binding correlated with ICAM-1 expression levels on stably transfected CHO clones. To analyze species cross-reactivity, 293T cells were transfected with either human or mouse ICAM-1. Analysis revealed specific binding of TP15-Fc to cells expressing human ICAM-1, but not to the murine homologue (Figure 4B).

### TP15-Fc induced significant ADCC against myeloma cell lines

**Figure 5:**
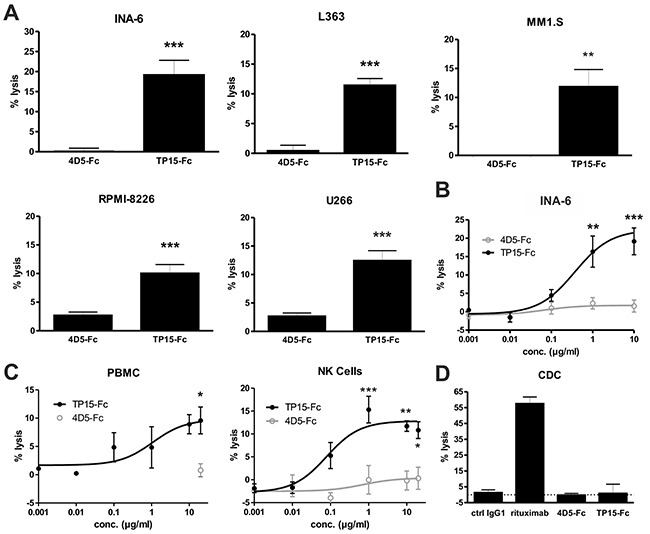
ADCC and CDC activity of TP15-Fc **(A)** ADCC was measured in ^51^Cr release assays with the plasma cell lines INA-6, L363, MM1.S, RPMI-8226, and U266. PBMC of healthy donors were incubated at a ratio of 80:1 with target cells and 10 μg/ml of the respective scFv-Fc fusion protein. **(B)** Concentration-dependent killing of INA-6 plasma cells with increasing antibody concentrations and human PBMC (80:1) is shown. **(C)** ADCC experiments were performed with mononuclear cells from one PCL and two myeloma patients containing 33 - 99 % CD138^+^ malignant plasma cells. PBMC (80:1; left graph) and purified NK cells (10:1; right graph) of healthy donors were used as effector cells to evaluate the potential of TP15-Fc to kill patient tumor cells. **(D)** CDC activity was tested with Raji Burkitt's lymphoma cells using human serum (50 μl/well) and 50 μg/ml of the indicated molecules. Rituximab was used as positive control. Experiments were done in triplicates at least twice. All graphs show % lysis ± SEM. (***) indicate p < 0.001, (**) p < 0.01 and (*) p < 0.05 of TP15-Fc *vs.* 4D5-Fc.

Fc-mediated effector functions of TP15-Fc were evaluated in standard ^51^Cr release assays. Using PBMC of healthy individuals at an effector-to-target (E:T) ratio of 80:1, TP15-Fc induced significant lysis of all five plasma cell lines tested when used at 10 μg/ml (Figure 5A). As shown for INA-6 plasma cells, significant dose-dependent killing was observed for TP15-Fc, but not for 4D5-Fc (Figure 5B). Concentration-dependent activity was further tested using either PBMC or purified NK cells of healthy donors with E:T ratios of 80:1 and 10:1, respectively, and patient-derived mononuclear cells containing 33 - 99 % CD138^+^ malignant plasma cells. As shown in Figure 5C, TP15-Fc induced dose-dependent lysis with PBMC and NK cells as effector cells. Notably, the EC_50_ value was more than 10-fold lower and in the pM range when purified NK cells instead of PBMC were used (EC_50__NK_ = 80 ng/ml *vs.* EC_50__PBMC_ = 1100 ng/ml, i.e. 645 pM *vs.* 8.9 nM). In contrast, only marginal ADCC activity was observed with TP15-Fc against HUVEC endothelial cells when NK cells were used, whereas 4D5-Fc was much more potent since Her2 is also expressed on these cells (Supplementary Figure 4B). Complement-dependent cytotoxicity (CDC) of TP15-Fc was evaluated with serum of healthy human donors and Raji and Daudi Burkitt lymphoma cells. In contrast to rituximab, no CDC activity was observed with TP15-Fc (Fig. 5D and Supplementary Figure 4C), which is most likely due to the A330L mutation that dramatically reduces C1q binding affinity [15]. Furthermore, TP15-Fc did not directly inhibit proliferation of INA-6, L363 and RPMI-8226 myeloma cells, as well as growth of HUVEC endothelial cells (Supplementary Figure 3, and 4D). Thus, the major anti-myeloma effector mechanism identified for TP15-Fc was ADCC with low reactivity observed against healthy human endothelial cells.

### TP15-Fc inhibited myeloma growth *in vivo*

**Figure 6:**
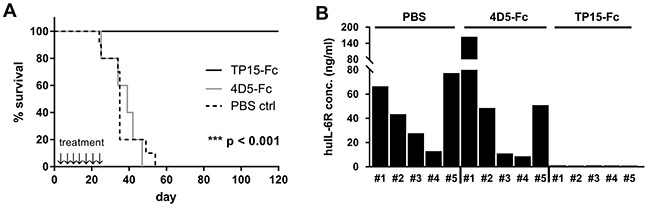
TP15-Fc prevents tumor engraftment in the INA-6 Tu1 xenograft model. **(A)** 48h after i.p. inoculation of 2.5×10^7^ INA-6.Tu1 cells in SCID/beige mice, mice were treated twice weekly either with vehicle (PBS control; dotted black line), 4D5-Fc (grey line) and TP15-Fc (black line), respectively. All mice received seven single doses (cumulative treatment dose: 1150 μg) by i.p. injection. Compared to the control mice (ten PBS and five 4D5-Fc-treated animals), which needed to be sacrificed within 55 days after tumor cell inoculation, INA-6.Tu1 cells did not engraft in the TP15-Fc-treated mice within 120 days (p < 0.001). ↓ = time point of i.p. injection. **(B)** Graph shows the human interleukin 6 receptor (huIL-6R) concentration in final sera of 5 mice per group measured by ELISA.

To investigate the *in vivo* potency of TP15-Fc, the INA-6.Tu1 xenograft model was used [30]. Five to ten SCID/beige mice were divided into three groups and treatment was started two days after tumor cell injection. Vehicle and 4D5-Fc treated mice developed tumors and needed to be sacrificed within 55 days (Figure 6A). Measurement of human interleukin 6-receptor (huIL-6R) in final sera of mice revealed huIL-6R concentrations between approx. 10 – 150 ng/ml that correlated with explanted tumor sizes (Figure 6B). In contrast, all animals receiving TP15-Fc survived until the end of the experiment without any sign of tumor growth and with no huIL-6R detectable in final sera (day 120; Figure 6). Thus, treatment with the ADCC-optimized TP15-Fc scFv-Fc fusion protein completely prevented INA-6.Tu1 plasma cell growth in this myeloma xenograft model.

## DISCUSSION

In the current report, a novel anti-human ICAM-1/CD54 scFv-Fc fusion protein (TP15-Fc) was identified by a cellular screening strategy intended to isolate antibodies for immunotherapy of MM from human phage libraries. Notably, TP15-Fc completely prevented myeloma cells growth in the INA-6.Tu1 *in vivo* model. *In vitro* TP15-Fc exerts its anti-myeloma activity predominantly via ADCC with human NK cells, an important effector mechanism of therapeutic mAbs.

By using highly diverse phage libraries and a cellular panning strategy with initial negative selection and three subsequent panning rounds with INA-6 and JK-6L plasma cell lines, phages could be isolated preferentially targeting cell surface structures on human myeloma cell lines. A whole-cell panning protocol and the flow cytometry-based isolation of tumor cells from mixtures with non-malignant cells can substantially enhance tumor specificity of the obtained phage antibodies [31–33]. Nevertheless, isolated phage antibodies were not exclusively myeloma-specific. Thus, developing even more stringent pre-absorption and panning strategies may further enhance the likelihood and frequency of phage antibodies with increased tumor selectivity [34, 35]. Today, phage display libraries with higher diversity are available, potentially allowing the identification of an even more versatile spectrum of binding specificities [36, 37]. Additionally, panning with freshly isolated patient-derived malignant plasma cells might further increase the possibility to identify novel antibodies against myeloma cell surface structures with potent anti-tumor activity since at least some antigens, e.g. CD307/FcRH5, might be lost upon cultivation [38].

Serological identification of antigens by recombinant expression cloning (SEREX) and serological proteome analysis (SERPA) may also display interesting strategies to identify novel myeloma-associated antigens [39, 40], but the majority of the identified epitopes/proteins targeted by the antibodies in patient sera often belonged to intracellular rather than to cell surface expressed structures that are accessible for therapeutic antibodies [41].

Phage PIII-15, chosen for further development, showed strong reactivity with myeloma and Burkitt's lymphoma cell lines, and patient-derived malignant plasma cells, while no or marginal binding to PBMCs, isolated T lymphocytes, monocytes or granulocytes of healthy donors was observed. By using the human scFv sequence of PIII-15, TP15-Fc was generated as a scFv-Fc fusion protein with an ADCC-optimized human IgG1 Fc. Therapeutically used human IgG1 mAbs exert their anti-tumor functions either by Fab- or Fc-mediated direct or indirect effector mechanisms. The engagement of the patient's immune effector cells has been suggested to be important, as the affinity of the Fc domain for activating and inhibitory Fc receptors (FcR) on immune cells can correlate with their therapeutic efficacy [12, 13, 42]. In this respect, ADCC and ADCP are relevant mechanisms of action of human IgG1 mAbs that are subject to optimization attempts [14, 43, 44]. For instance, the S239D and I332E mutations, also integrated into the Fc domain of TP15-Fc, were reported to substantially enhance ADCC activity by improving the affinity for the activating FcγRIIIa [15]. Indeed, TP15-Fc was capable to induce potent ADCC with human PBMC and isolated NK cells against different plasma cell lines and patient-derived malignant plasma cells with EC_50_ values in the low nM range. Especially, NK cells are potent effector cells and represent a powerful cell population for anti-myeloma immunotherapy. Some IMiDs, i.e. thalidomide and lenalidomide, as part of the current myeloma treatment strategies, are known to stimulate NK cell activation and may augment anti-tumor activity of therapeutic mAbs or Fc-containing antibody-derived molecules via ADCC [45, 46]. For daratumumab and elotuzumab, the two anti-myeloma IgG1 mAbs that have been recently approved for MM therapy, ADCC activity has been described as important mechanism of action and high efficacy is seen in combination with PIs and IMiDs in myeloma patients [9, 47–49]. Although Fc-engineered mAbs directed against HM1.24 and CD40 showed potent pre-clinical activity, currently no ADCC-optimized antibodies are evaluated in clinical studies for MM therapy [20, 27].

Human ICAM-1/CD54 was identified as target antigen of TP15-Fc. ICAM-1 is a 90 kDa trans-membrane glycoprotein, consisting of five Ig-like extracellular domains and thus belonging to the Ig superfamily [50, 51]. Its expression is constitutively low on leukocytes, platelets, fibroblasts, endothelial and epithelial cells under non-inflammatory conditions, which is consistent with the binding pattern observed for TP15-Fc. Since ICAM-1 is an important adhesion molecule playing a key role in leukocyte extravasation during states of inflammation, it was also associated with metastatic progression of cancer [52]. In myeloma, overexpression of ICAM-1 was negatively correlated with patient survival, and ICAM-1-CD18 interactions seem to be involved in macrophage-induced drug resistance [53, 54]. The first ICAM-1 antibody tested against human tumor cells was UV3 developed by the group of Ellen Vitettta in 1993 [55]. Potent anti-tumor activity against different tumor entities, e.g. Burkitt's lymphoma, was observed for the mouse IgG2 antibody and the chimeric cUV3 in preclinical *in vitro* and *in vivo* studies [56–58]. Enlimomab (BIRR1/R6.5), another anti-human ICAM-1 antibody, was also tested in clinical trials to treat rheumatoid arthritis, prevent acute rejection of renal transplants and in acute stroke patients, and showed some clinical improvements for arthritis as well as stroke patients and importantly good tolerability [59, 60]. The first human anti-ICAM-1 IgG1 mAb BI-505 was recently evaluated in clinical trials for patients with relapsed/refractory MM and smoldering myeloma [61, 62]. Even though future clinical development was stopped, BI-505 was mostly well tolerated. The maximum tolerated dose was not reached up to 20 mg/kg while ICAM-1 epitopes on patients’ myeloma cells were saturated already at 10 mg/kg. Adverse events were mostly infusion-related and were in general mild to moderate [61]. Thus, pronounced toxicity of anti-ICAM-1 antibodies were not reported – suggesting a therapeutic window for TP15-Fc antibody therapy in myeloma.

As for TP15-Fc, also for UV3 and BI-505 Fc-mediated effects were reported to be crucial for their efficacy [57, 63]. For instance, potent macrophage-dependent anti-myeloma activity was described for BI-505 besides the induction of programmed cell death. In contrast, ADCC and the recruitment of human NK cells is most likely the major effector mechanism of TP15-Fc. These different modes of action may be due to different epitopes on ICAM-1/CD54 bound by the individual antibodies and may additionally rely on the mutations introduced in the Fc part of TP15-Fc to improve the FcγR binding of the molecule. Future studies are required to address if the expression pattern of ICAM-1/CD54 may cause higher toxicity if an Fc-engineered compared to a wild type mAb is used. Thus, we currently work on the generation of fully human IgG1 antibody variants with differentially modified Fc parts and on more advanced animal models (i.e. an orthotopic mouse model with fluorescent tumor cells) to address these questions. Furthermore, combination treatments with IMiDs and PIs will be tested since treatment regimen including these agents and a mAb proved to be very efficient in myeloma patients and led to the approval of daratumumab and elotuzumab. Since ICAM-1/CD54 is also believed to be important for the interaction of malignant plasma cells with the bone marrow microenvironment, we will define the exact binding epitope of TP15-Fc and investigate if there is an impact on the interaction with BMSC or other ligand-expressing cells and if this has a therapeutic impact.

In summary, phage display technology combined with a cellular panning strategy led to the identification of the novel human ICAM-1 antibody TP15-Fc. This antibody exerts its anti-myeloma activity by efficiently recruiting NK cells for ADCC. Although caution is required in translating observations from animal models into the clinical situation in man, the complete control of human myeloma cell growth in the SCID xenograft model is encouraging to further evaluate TP15-Fc for MM immunotherapy.

## MATERIALS AND METHODS

### Cell separation

Mononuclear cells from myeloma patients as well as PBMC and granulocytes/PMN from healthy donors were isolated as described previously [16]. Samples were taken after receiving donors’ written informed consent. All experiments were in accordance with the Declaration of Helsinki and approved by the Ethics Committee of the Christian-Albrechts-University, Kiel, Germany.

NK cells, monocytes, B and T lymphocytes were enriched by negative selection using MACS technology (Miltenyi Biotec, Bergisch Gladbach, Germany). Viability and purity had to be > 95% as verified by flow cytometry before use in experiments.

### Culture of eukaryotic cells

Jurkat, Ramos, Raji, Daudi, and CHO-K1 cells were obtained from the German Collection of Microorganisms and Cell Cultures (DSMZ, Braunschweig, Germany), whereas ARH-77, CEM, HEL, HL-60, K562, KG-1a and RPMI-8226 were purchased from the American Type Culture Collection (ATCC, Manassas, VA, USA). SEM, L363, MM1.S, and U266 were kind gifts from J. Greil (Erlangen, Germany), V. Diehl (Cologne, Germany), Y.T. Tai (Boston, MA, USA) and K. Nilsson (Uppsala, Sweden), respectively. ARH-77, CEM, Daudi, HEL, Jurkat, K562, L363, MM1.S, Raji, RPMI-8226, U266 cells were cultured in RPMI 1640-Glutamax-I medium containing 10% FCS, 1% penicillin and streptomycin (R10+; all Life Technologies, Carlsbad, CA, USA). The human plasma cell lines INA-6, INA-6.Tu1 and JK-6L were established in our laboratory [30, 64]. JK-6L were cultivated in R10+, while INA-6 and INA-6.Tu1 were cultured in R10+ supplemented with 2.5 ng/ml recombinant human IL-6 (Life Technologies). Ramos cells were kept in RPMI 1640-Glutamax-I medium plus 20% FCS, penicillin and streptomycin. SEM, KG-1a and HL-60 were maintained in Iscove's MDM (Life Technologies) with 10% or 20% FCS and antibiotics. Human umbilical vein endothelial cells (HUVEC) were obtained from Lonza and kept in EGM-Plus medium (Lonza, Walkersville, MD, USA). Lenti-X 293T (Clontech, Mountain View, CA, USA) and CHO-K1 cells were cultured in DMEM (Life Technologies) containing 10% FCS and antibiotics (D10+).

### Transfection of CHO-K1 and Lenti-X 293T cells

To transiently transfect Lenti-X 293T cells with human (pCMV6-XL5 human ICAM-1 (untagged); NM_000201.1) or mouse ICAM-1 (pCMV6-AC-GFP mouse ICAM-1 (GFP-tagged); NM_010493.2; both OriGene Technologies, Rockville, MD, USA), Lipofectamine LTX (Life Technologies) transfections were performed according to manufacturer's instructions. Cells were maintained in D10+ for 72h prior flow cytometric analysis. Stable transfection of CHO-K1 cells was carried out as described in the supplements.

### Panning of the phage display libraries

The two human synthetic scFv libraries Tomlinson I and J with a size of 1.47×10^8^ and 1.37×10^8^, respectively, were purchased from the MRC HGMP Resource Centre, Cambridge, UK. Phage stocks with 10^12^-10^13^ phages/ml were prepared according to the manufacturer's instructions and were utilized for panning. All cells and reaction tubes were pre-incubated with 2% (w/v) non-fat dry milk powder (Bio-Rad, Munich, Germany) in PBS (NM-PBS; Life Technologies) for at least 30 min. 5×10^12^ phages were incubated with intact cells for 2h on a roller incubator at 4°C. First the libraries were step-wise pre-absorbed with freshly isolated granulocytes and T lymphocytes from healthy individuals (50×10^6^ cells each), then incubated with a mixture of 2.5×10^6^ JK-6L cells and 50×10^6^ T lymphocytes. Subsequently, T cells were eliminated with CD3 microbeads and LD depletion columns (Miltenyi Biotec). The CD3-negative fractions were stained with CD138-PE (Beckman Coulter, Krefeld, Germany) and CD3-FITC (BD Biosciences, Heidelberg, Germany; clone SK7) to sort the CD138^+^ plasma cells with a FACS Canto analyzer and Diva software (BD Biosciences). Phages bound to plasma cells were eluted with 1 mg/ml trypsin (Sigma-Aldrich, Munich, Germany) in PBS for 10 min at RT and used to re-infect *E. coli* TG1. The protocol was followed as recommended for panning with a complex antigen and two additional rounds with 2.5×10^6^ INA-6 (round 2) and JK-6L cells (round 3) were carried out. Phage precipitations were done with 20% polyethylene glycol 6000 in 2.5 M NaCl (PEG/NaCl; Carl Roth, Karlsruhe, Germany) prior titration and storage at 4°C.

### Production of monoclonal phage antibodies

To isolate monoclonal phages, individual colonies were grown in 96-well-plates and infected with helper phage according to standard procedures. Bacteria were pelleted and supernatants were tested in whole-cell ELISA experiments. Monoclonal phages with desired binding properties were subsequently produced in 100 ml *E. coli* cultures followed by PEG/NaCl precipitation and titration.

### Flow cytometric analyses

To analyze cell surface binding, 0.5×10^6^ cells were incubated with 5×10^10^ phages or scFv-Fc fusion protein at the indicated concentration in PBS supplemented with 1% BSA and 0.1% NaN_3_ (PBA buffer; Sigma-Aldrich) on ice for 60 min. Bound phages were detected with an anti-fd bacteriophage antibody (Sigma-Aldrich), which had been labeled with DyLight 488 Microscale Antibody Labeling Kit (Thermo Fisher Scientific, Waltham, MA, USA). The scFv-Fc fusion proteins were detected with a Penta-His Alexa Fluor 488 Conjugate (Qiagen, Hilden, Germany) or goat F(ab)2 anti-human Fcγ (IgG)-FITC and -PE (Beckman Coulter; final dilution for all 1/20 in PBA), respectively. Samples containing FcR carrying immune cells, e.g. NK cells and monocytes, were pre-incubated for 15 min with PBA buffer supplemented with human immunoglobulin (Intratect, Biotest Pharma, Dreieich, Germany) in a final concentration of 500 μg/ml to prevent unspecific binding of the scFv-Fc fusion proteins via their Fc domain. Subsequently, samples were directly stained with DyLight 488-labeled TP15-Fc or 4D5-Fc or indirectly stained with the Penta-His Alexa Fluor 488 Conjugate (Qiagen). All samples were measured on a Navios flow cytometer and analyzed with Kaluza software (Beckman Coulter).

### Whole cell ELISA

96-well flat-bottom plates were blocked with NM-PBS prior incubation of 0.5×10^6^ cells with 50-100 μl phage-containing *E. coli* TG1 supernatant or 5-25×10^10^ PEG/NaCl precipitated phages/well on a plate shaker for 1h at RT. Plates were washed five times with 0.1% NM-PBS prior incubation with an HRP-labeled anti-M13 antibody (GE Healthcare, Solingen, Germany) diluted 1/2000 in 1% NM-PBS. Plates were washed as described and 100 μl ABTS solution/well (Roche, Rotkreuz, Switzerland) was added. Absorbance (405/670 nm) was measured with a Spectra Rainbow Reader (Tecan, Männedorf, Switzerland).

### ScFv-Fc fusion protein construction

To allow scFv fusion and cloning into the pSec-IgG1-Fc-prot-eng vector [65], the scFv sequence was amplified using primers LMB3 forward (5’ CAATTTCACACAGGAAACAGCTATGAC 3’) and *Not*I *Psp*OMI reverse (5’ TTCGATCGGGCCCCCTGCGGCCGCCCGTTTGATTTCCACC 3’). PCR was performed with *Pwo* DNA Polymerase. The PCR product was extracted from agarose gel with NucleoSpin Gel and PCR Clean-Up kit (Machery-Nagel, Dueren, Germany). Vector and PCR product were digested with *Sfi*I/*Not*I and *Sfi*I/*Psp*OMI (NEB, Ipswich, MA, USA), respectively, and cloned by using standard procedures. The amino acids exchanged in the Fc domain (S239D/I332E/A330L; ref. 15) enhance FcR binding and considerably reduce C1q binding of the resulting Fc-engineered scFv-Fc fusion protein TP15-Fc. The 4D5 scFv (anti-HER2) was used to design the control molecule 4D5-Fc [66]. Both fusion proteins contained a 6xHis-tag for purification and detection. The correct sequences of the final constructs were verified by Sanger sequencing.

### Expression and purification of scFv-Fc fusion proteins

The recombinant proteins were expressed in Lenti-X 293T cells by calcium phosphate transfection and purified by affinity chromatography with protein A (GE Healthcare) and Ni-NTA beads (Qiagen). Gel filtration of the proteins were performed on an ÄKTA purifier system with Unicorn 5.1 software (GE Healthcare) using PBS as running buffer at a constant flow rate of 0.1 ml/min. 200 μg protein was loaded on a Superdex 200 10/300 GL column (GE Healthcare). Ovalbumin and aldolase of the Gel Filtration HMW Calibration Kit (GE Healthcare) were used as molecular weight controls. Quantifications were done by capillary electrophoresis (Experion Pro260 kit; Bio-Rad). Purity and molecular masses of TP15-Fc and 4D5-Fc (5 μg protein each) were analyzed on Coomassie gel by using standard procedures.

### Chromium release assay

ADCC and CDC were analyzed in standard ^51^Cr release assays as previously described [16]. Briefly, 5 000 target cells were incubated with the indicated molecules and the respective human effector cells or 50 μl human serum at 37°C for 3h. PBMC and human NK cells of healthy individuals were applied at E:T ratios of 80:1 and 10:1, respectively.

### Animal model

TP15-Fc was tested in the INA-6.Tu1 xenograft model [30]. 8 weeks old female SCID/beige mice (Charles River, Sulzfeld, Germany) were injected intraperitoneally (i.p.) with 25×10^6^ INA-6.Tu1 plasma cells 48h prior start of treatment. Groups of 5 or 10 mice were treated either with TP15-Fc, 4D5-Fc or vehicle control (PBS) twice weekly i.p. for a total of seven doses. Cumulative treatment dose was 1.15 mg scFv-Fc fusion protein/animal. The experiments were performed according to the animal experimental guidelines of the responsible local authorities and along with the German Animal Protection Law. Human IL-6 receptor/CD126 concentration in final mice sera was analyzed with CD126 ELISA kit (Diaclone, Besançon, France) according to the instructions.

### Data processing and statistical analyses

Data were analyzed with GraphPad Prism 5.0 (GraphPad Software Inc., San Diego, CA, USA). Curves were fitted using a nonlinear regression model with a sigmoidal dose response (variable slope). Group data are reported as mean ± SEM. Differences between groups were analyzed by one- or two-way ANOVA with Bonferroni post-test or by Mann-Whitney two-tailed t-test. Survival curves were analyzed with log-rank test. Significance was accepted with *p* < 0.05.

### Homology modeling

The homology model for TP15-Fc was calculated using YASARA Structure software (YASARA Biosciences, Vienna, Austria) after removing secretion leader and tags.

## SUPPLEMENTARY MATERIALS FIGURES AND TABLE



## Abbreviations

ADCC: antibody-dependent cell-mediated cytotoxicity
ADCP: antibody-dependent cellular phagocytosis
BM: bone marrow
BMSC: bone marrow stromal cells
CDC: complement-dependent cytotoxicity
ELISA: enzyme-linked immunosorbent assay
FcR: Fc receptor
ICAM-1: intercellular adhesion molecule-1
Ig: immunoglobulin
IMiD: immunomodulatory drug
mAb: monoclonal antibody
MM: multiple myeloma
PBMC: peripheral blood mononuclear cells
PCL: plasma cell leukemia
PI: proteasome inhibitor
scFv: single-chain fragment variable
