# Supplementary material for: A novel Fc-engineered human ICAM-1/CD54 antibody with potent anti-myeloma activity developed by cellular panning of phage display libraries
Source: Oncotarget. 2017 Sep 5;8(44):77552–66. doi: 10.18632/oncotarget.20641 (PMC5652799; doi:10.18632/oncotarget.20641)
Supplement: Supplementary file 1 [file oncotarget-08-77552-s001.pdf]

A novel Fc-engineered human ICAM-1/CD54 antibody with potent anti-myeloma activity developed by cellular panning of phage display libraries

SUPPLEMENTARY MATERIAL

Stable transfection of CHO-K1 cells

CHO-K1 cells were transfected with Lipofectamine 2000 (Life Technologies) according to manufacturer's instructions. To generate stable transfected cells with pCMV6-AC CD38 (NM_001775.2) or pCMV6-Entry CD56 (NM_181351.3) / CD70 (NM_001252.2) / CD126 (NM_000565.2) / CD319 (NM_021181.3) / CD333 (NM_000142.2), neomycin (Geneticin, 500 µg/ml; Life Technologies) was added 72h post transfection. Cells transfected with pCMV6-XL4 CD138 (NM_002997.4), pCMV6-XL5 CD40 (NM_001250.3) or CD54 (NM_000201.1; all OriGene Technologies) needed to be co-transfected with pSecTag2/HygroC (Life Technologies) in a 10:1 ratio to generate stable transfectants by hygromycin B (500 µg/ml; Life Technologies) selection. Single clones were isolated by limiting dilution and kept in D10+ supplemented with hygromycin B or neomycin and tested by flow cytometry.

Antibodies used for the characterization of stable CHO transfectants

IgG (mouse)-FITC (isotype control), IgG (mouse)-PE (isotype control), CD38-FITC, CD40-PE, CD54-FITC, and CD56-PE were used from Beckman Coulter (Krefeld, Germany), mouse IgG2a PE-conjugated isotype control, anti-human CRACC-PE, anti-human IL-6R-PE, and anti-human FGFR3-PE from R & D Systems (Minneapolis, MN, USA). Anti-mouse IgG F(ab)2 fragment-FITC and mouse anti-human CD70 were purchased from Sigma-Aldrich (Munich, Germany) and AbD Serotec (Puchheim, Germany), respectively. TIB92 (mouse IgG2a) hybridoma supernatant was directly used as a control.

Cell growth inhibition experiments (MTS assay)

To evaluate the direct effects of TP15-Fc, target cells were seeded at a density of 2×10^4 cells/200 µl per well in 96-well-plates and incubated in the presence of 10 µg/ml of the respective antibody at 37 °C for 72 h. Vital cell mass was measured by a colorimetric method using MTS cell proliferation assay according to manufacturer's protocol (Promega). 4D5-Fc was used as control. Percent growth of antibody-treated cells was calculated compared to untreated cells.

Flow cytometric analyses to determine specific antigen binding site

ICAM-1 expression was quantified using saturating concentrations of 500 µg/ml of TP15-mFc and 4D5-mFc containing a murine IgG1 Fc (unpublished), respectively, and $0{,}5 \times 10^6$ indicated cells per sample with the QIFIKIT according to the manufacturer's instructions (DAKO, Denmark). Samples were analyzed on a flow cytometer (Navios; Beckman Coulter, Fullerton, CA).

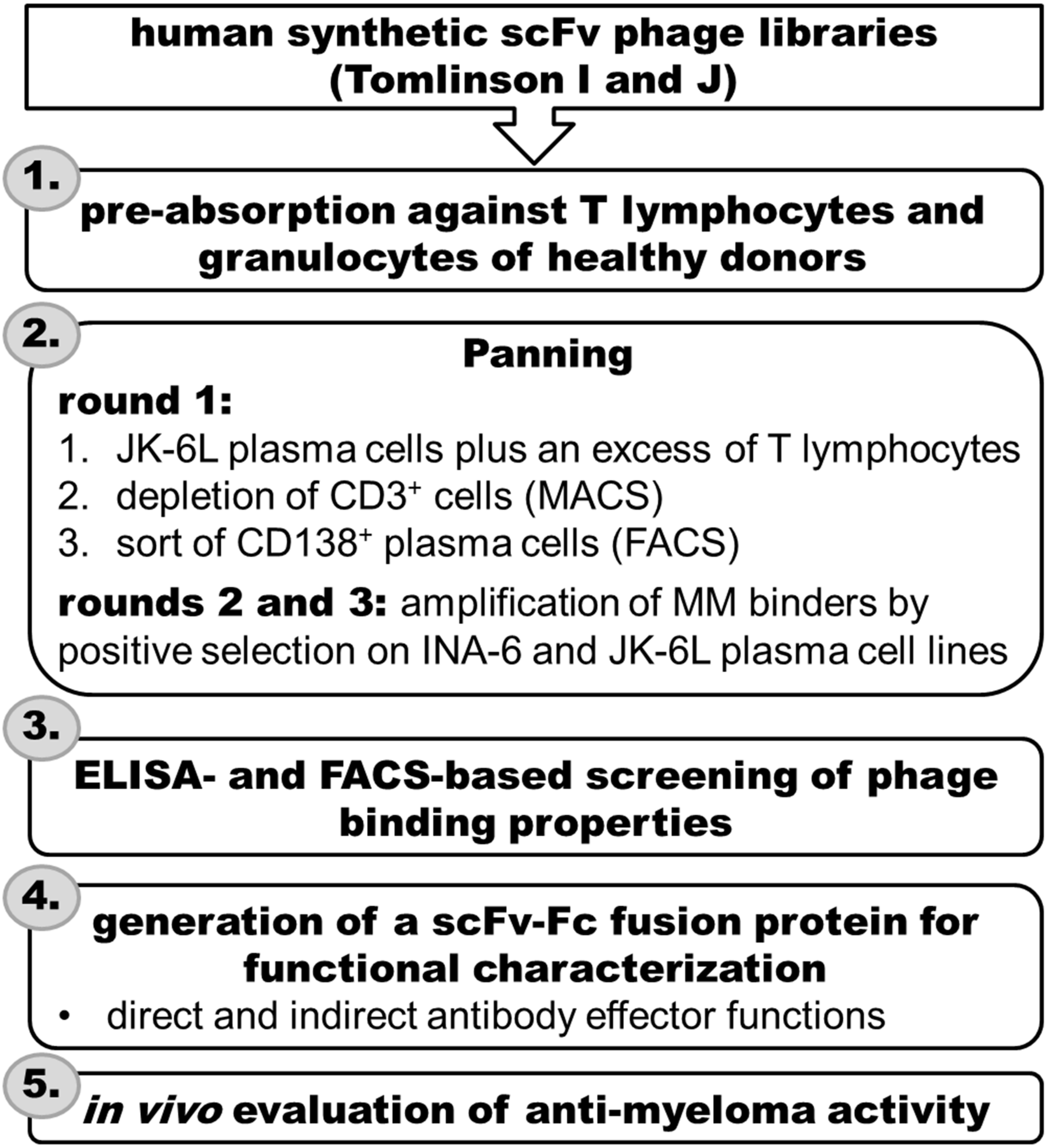

Supplementary Figure 1: Cellular panning strategy applied for the two Tomlinson phage display libraries. Overview over the individual steps employed to amplify and characterize myeloma cell binding phage antibodies.

A

stable CHO clone/antigen	MFI antigen	PIII-15 binding
CD38	16.6	-
CD40	31.1	-
CD54 / ICAM-1	6.2	+
CD56 / NCAM	59.8	-
CD70	13.3	-
CD126 / IL-6R	2.1	-
CD138	3.8	-
CD319 / CS1	5.1	-
CD333 / FGFR3	4.8	-

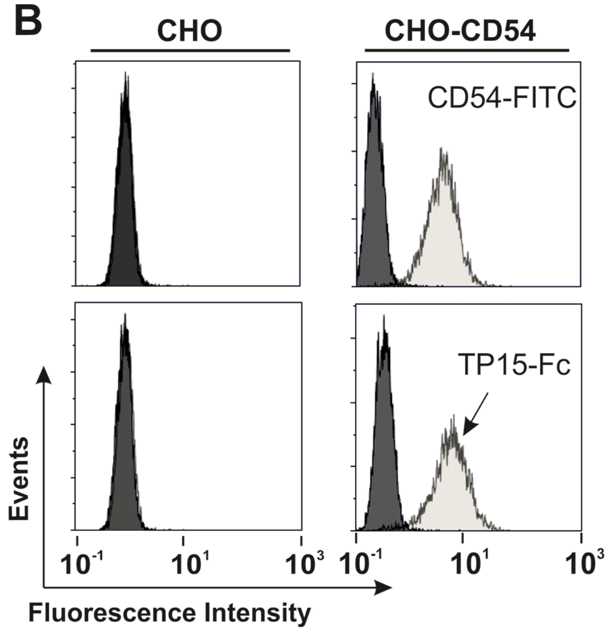

Supplementary Figure 2: Binding of TP15-Fc to stable CHO transfectants expressing selected myeloma antigens. (A) List of the generated stably transfected CHO-K1 cell lines expressing individual myeloma antigens, their mean fluorescence intensity (MFI) detected by commercially available antibodies and positive (+) or negative (-) binding observed with PIII-15/TP15-Fc. **(B)** Flow cytometric analyses of non-transfected and stably CD54-transfected CHO cells revealed strong binding of 10 µg/ml of the scFv-Fc fusion protein TP15-Fc (light grey, lower histogram). Binding was comparable with that obtained by 10 µl CD54-FITC antibody of Beckman Coulter (light grey, upper histogram). 4D5-Fc and mouse IgG isotype control were used as controls (black). TP15-Fc and 4D5-Fc were detected with a FITC-labeled anti-human IgG secondary antibody.

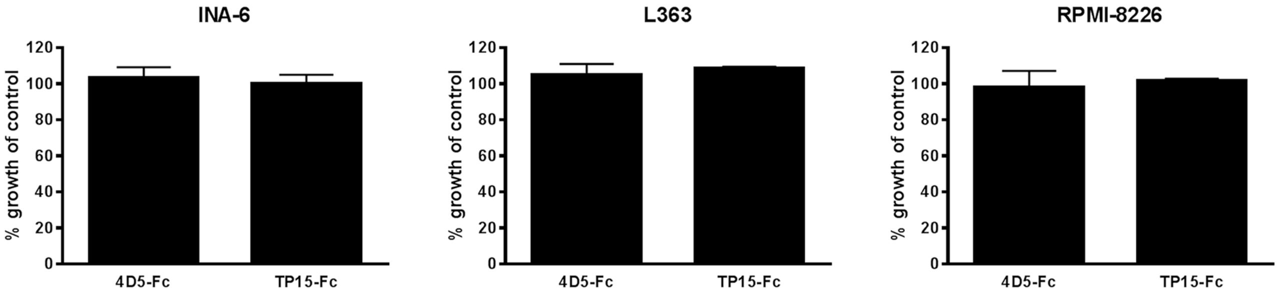

Supplementary Figure 3: TP15-Fc does not inhibit myeloma cell proliferation. Treatment of INA-6, L363 and RPMI-8226 myeloma cells with 10 µg/ml TP15-Fc for 72 h did not inhibit cell growth as compared to control molecule 4D5-Fc. Percent growth of control was calculated versus untreated cells. Results of 3 independent experiments with triplicates are shown as mean ± SEM.

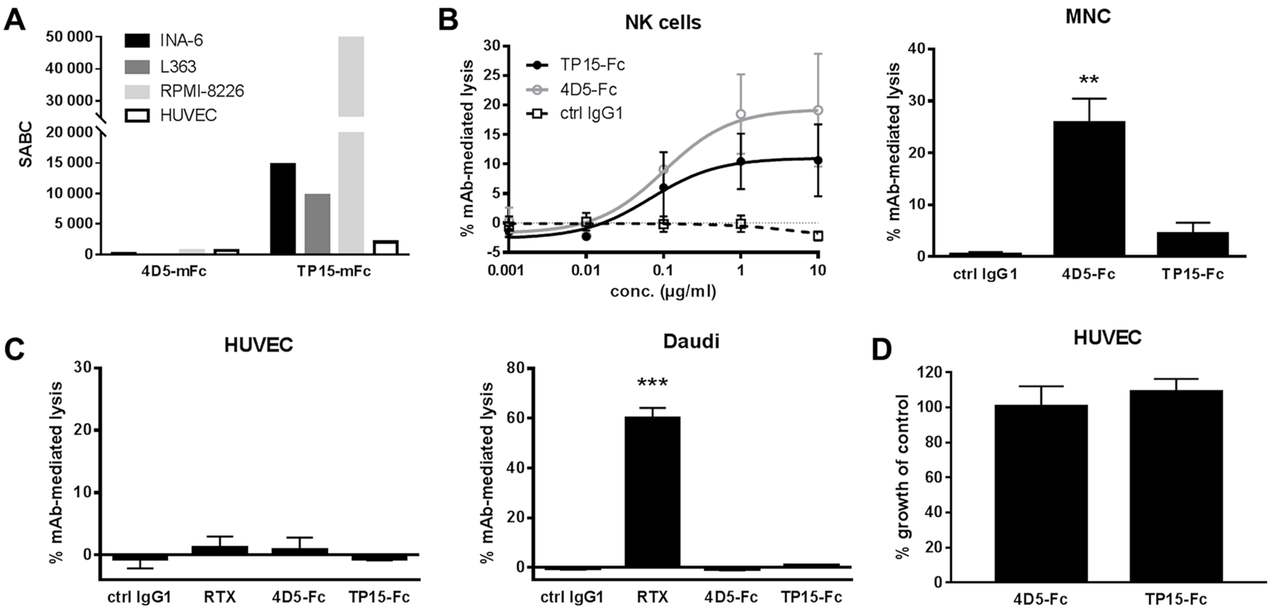

Supplementary Figure 4: Binding of TP15-Fc to HUVEC endothelial cells and the functional consequences thereof. (A) Quantative flow cytometry analyses were performed with 0.5x10^6 of the indicated cells by using 500 µg/ml of antibody carrying a mouse Fc domain (mFc). Specific antigen binding sites per cell (SABC) were quantified according to the manufacturer. **(B)** ADCC experiments were performed with isolated human NK cells at an effector-to-target (E:T) ratio of 10:1 (left graph) using increasing antibody concentrations, or with mononuclear cells (MNC) as effector cells (E:T ratio 80:1; right graph) and 10 µg/ml antibody. **(C)** For CDC experiments 0.5x10^6 target cells, freshly drawn human serum and 10 µg/ml of the indicated antibodies were used. Daudi cells and rituximab (RTX) served as positive control to show the functionality of the assay (right graph). ADCC and CDC experiments were performed using effector cells/ serum from two different donors in standard chromium release assays. ** $p < 0.01$, *** $p < 0.001$. **(D)** Proliferation inhibition was investigated by MTS assay using 5.000 cells and 100 µg/ml TP15-Fc and 4D5-Fc, respectively. Percent growth inhibition was calculated compared to untreated cells of three independent experiments.

Supplementary Table 1: Enrichment of phages during panning

	Tomlinson I				Tomlinson J			
	Input titer[a]	Output titer[a]	Ratio [b]	EF [c]	Input titer[a]	Output titer[a]	Ratio [b]	EF [c]
pre-abs.	5 x 1012	7 x 1011	0.14	-	5 x 1012	8 x 1011	0.16	-
round								
1st	7 x 1011	1 x 103	1.4 x 10-9	-	8 x 1011	6 x 103	7.5 x 10-9	-
2nd	9 x 1012	1 x 108	1.1 x 10-5	7857	2 x 1013	2 x 108	1 x 10-5	1333
3rd	3 x 1013	2 x 108	6.6 x 10-6	0.6	1 x 1013	2 x 108	2 x 10-5	2

pre-abs. = pre-absorption.[a] input and output titers are given in cfu = colony forming unit per ml. [b] ratio = output titer/input titer. [c] EF = enrichment factor defined as ratio (n+1)/ratio (n).
